# Molecular pathogenetic mechanisms and new therapeutic perspectives in anthracycline-induced cardiomyopathy

**DOI:** 10.1186/1824-7288-35-37

**Published:** 2009-11-20

**Authors:** Giuseppe Distefano

**Affiliations:** 1 Department of Pediatrics - Division of Neonatology and Pediatric Cardiology, University of Catania, Catania, Italy

## Abstract

Anthracyclines are among the most powerful drugs for the treatment of oncologic diseases both in childhood and in adulthood. Nevertheless, their major antineoplastic efficacy can be seriously impaired by collateral toxic cardiac effects causing cardiomyopathy with chronic heart failure that is refractory to conventional medical therapy.

This article reports possible subcellular molecular alterations of anthracycline-induced cardiomyopathy (reactive oxygen species formation, apoptosis, inflammatory signalling, altered expression of cardiomyocytes specific genes, etc) and indicates some new therapeutic perspectives resulting from a better understanding of the molecular pathogenetic mechanisms.

## 

Today anthracyclines are among the most powerful drugs used for the treatment of oncologic diseases both in childhood and adulthood. Nevertheless their major antineoplastic efficacy can be seriously impaired by collateral toxic effects causing profound alterations in cardiac muscle. These effects can be associated to acute clinical manifestations, occurring within 24 hours from the beginning of treatment, such as hyperkinetic arrhythmias and/or reversible heart failure (myocarditis-pericarditis syndrome); subacute manifestations, occurring after weeks or months (up to 30 months), leading rapidly to progressive heart failure and 60% mortality; chronic manifestations, occurring 4-20 years after the treatment, with progressive irreversible cardiac insufficiency [1]. The most interesting aspects are connected to late chronic cardiotoxicity that is particularly insidious. It has a long term asymptomatic course or presents slight electrocardiographic and/or echocardiographic anomalies that later evolve into chronic cardiomiopathy, dilated type in adulthood and restrictive-dilated in childhood, that is refractory to medical treatment [2]. Another peculiar feature of chronic anthracycline cardiotoxicity is that it is strictly linked to drug cumulative dose. Indeed, the incidence of anthracycline - induced cardiomyopathy (AIC) and heart failure increases from 7% of cases for total doses of 550 mg/m^2^/bs, to 15% for 600 mg/m^2^/bs and 30-40% for 700 mg/m^2^/bs [3].

Pathological studies on experimental animal models and human endomyocardial biopsies have shown that AIC is characterized by histological alterations consisting in multiple areas of interstitial fibrosis associated with the presence of cardiomyocytes with vacuolar degeneration or compensatory hypertrophy. Necrotic cardiomyocytes with histiocytic infiltration, and stromal oedema with myocardial fibers dissociation can also be observed. Electron microscopy revealed that the damage caused by anthracyclines to cardiomyocytes appears as loss of myofibrils, distention of sarcoplasmic reticulum, mitochondrial swelling, increased lysosomal number and disorganization of nuclear chromatine [4-6].

In order to explain these alterations, numerous pathogenetic mechanisms have been proposed [6], and three seem to be the most important: free radical release secondary to the binding of anthracyclines to intracellular iron, interaction with nuclear and mitochondrial DNA, and gene activation with biochemical transduction signals inducing apoptosis [7,8].

Free radicals cardiac toxicity can be caused by direct damage of the mitochondrial respiratory chain with consequent decrease in energy production, due to phosphorilative processes impairment, and reduction of cardiomyocytes following the release of pro-apoptotic factors. Both effects lead to altered systolic function [7] (Figure 1). Further harmful actions of free radicals are associated with membrane lipid peroxidation and cytoskeleton protein oxidation. These events cause the dysfunction of membrane and sarcotubular ATP-ases systems with consequent intracellular calcium increase, and altered sarcomeric motility impairing the relaxing ability of cardiomyocytes that induces deficient diastolic function [9] (Figure 1). Initially, the loss of contractile elements is compensated by the hypertrophy of surviving cardiomyiocytes, thus masking the alteration of systolic function. On the other hand, cardiac cells have a low content of antioxidant systems and can be easily damaged by oxidative stress.

**Figure 1:**
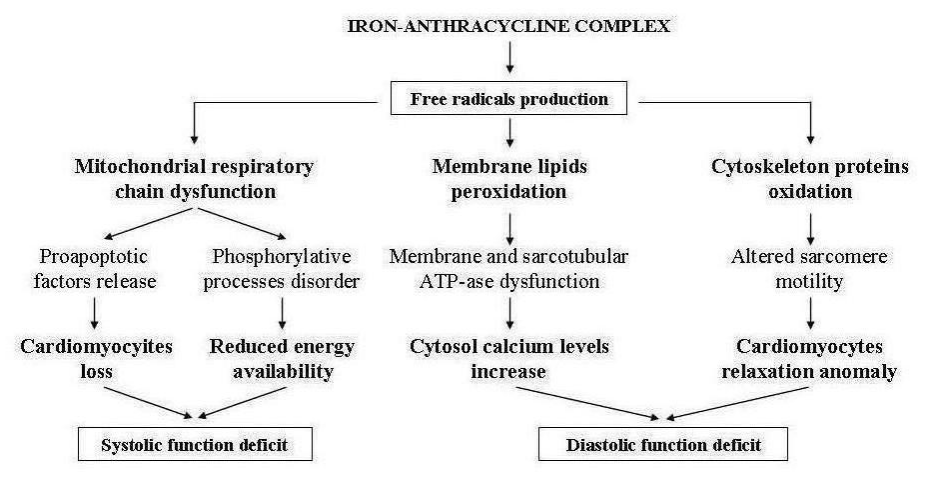
**Role of free radicals in the pathogenesis of anthracycline cardiomyopathy**.

Furthermore, interferences with nuclear DNA can inhibit protein synthesis and cardiac tissues growth and down-regulate contractile, sarcotubular and cytosolic proteins. Moreover, these interferences can determine the re-expression of genes that are active during the embrio-fetal period when they code the synthesis of both pro-apoptotic factors and enzymatic and functionally immature muscular proteins. Conversely, interferences with mitochondrial DNA mainly affect the mitochondrial respiratory chain function that can be seriously impaired by the inhibition of cardiolipin, a phospholipid which plays a crucial role in the regulation of cardiac energetic processes. Alterations of the subunits of mitochondrial respiratory complexes can also cause the release of cytochrome c, which can determine cardiomyocytes apoptosis by activating caspases and metalloproteinases enzymatic system. [10,11] (Figure 2). All these processes involving both nuclear and mitochondrial DNA may be linked to anthracycline alcoholic metabolites, and their negative effects on cellular energetic metabolism, protein synthesis and myocardial tissues development can explain the different clinical evolution of AIC in adulthood and in childhood [12]. In adults the loss of cardiomyocytes induced by apoptosis, together with the inhibition of compensatory hypertrophy and with the energetic deficit, can cause ventricular dilatation resulting from the thinning of ventricular walls and the reduction of contractile force, leading to the development of dilated cardiomyopathy. In children the dilatation of ventricular cavities can be associated with a restrictive hemodynamic status following reduced cardiac dimensions caused by the slower development of myocardial mass. This reduces ventricular compliance and thus determines restrictive-dilated cardiomyopathy [13,14] (Figure 3). The subsequent evolution of these two types of AIC is characterized by inexorable progressive deterioration of cardiac function leading to severe and refractory heart failure with fatal exitus.

**Figure 2:**
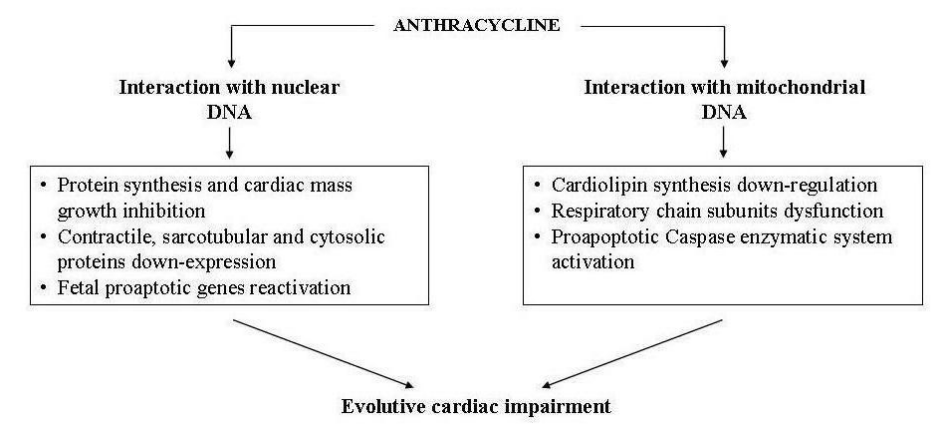
**Pathogenetic mechanisms of anthracycline cardiomyopathy**.

**Figure 3:**
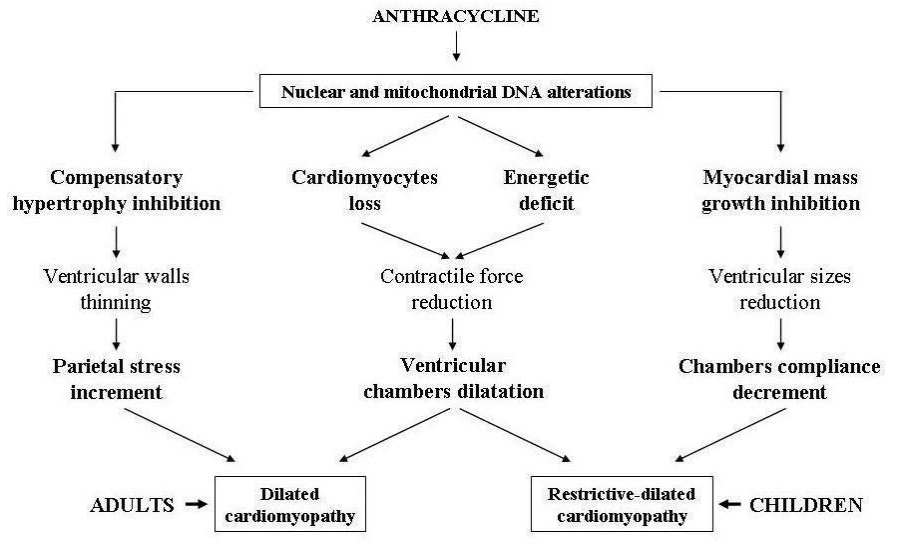
**Different haemodynamic evolution of anthracycline cardiomyopathy in relation to patient's age**.

At this point, the mechanisms responsible for this negative evolution of AIC can overlap others that progressivley exacerbate all dilated chronic cardiomyopathies (regardless of their aetiology) and are strictly influenced by the neuro-hormonal response triggered by the chronic cardiac contractility deficit. This response activates the adrenergic and renin-angiotensin-aldosterone system and the release of catecholamines, angiotensin and aldosterone. Excess of circulating amines down-regulates cardiac adrenergic receptors and reduces the inotropic response to adrenergic signals thus further impairing contractile deficit. Peripheral vasoconstriction and hydro-saline retention are induced respectively by angiotensin and aldosterone, and increase pre and the post-load thus jeopardizing cardiac performance and giving rise to a vicious circle with progressive cardiac dysfunction [15,16] (Figure 4). Moreover, at the genesis of this dysfunction, besides the above-mentioned negative haemodynamic effects, modifications of the ultrastructure of cardiac muscle induced by the same adrenergic amines, aldosterone and angiotensin may be involved. Recent studies on molecular cardiology showed that these substances can be released inside the cardiac muscle after "cardiac mechano-receptors stimulation" due to bio-mechanical stress induced by volume and/or pressure overload secondary to heart failure [17]. This intra-myocardial neuro-hormonal response is far greater than that of the circulatory district and, like catecholamines, angiotensin and aldosterone, includes cardiomyocytes release of endothelin, cytokines and peptides growth factors (Figure 5). These molecules, acting in autocrine and paracrine fashion in the same cardiomyiocytes and in the surrounding tissues, determine profound modifications of ultrastructural cardiac architecture with functional alterations at the level of the finest subcellular mechanisms. These ultrastructural changes constitute the basis of the so called "myocardial remodelling" a biological process, peculiar to the natural history of chronic heart failure, characterized by several cellular and molecular events consisting of hypertrophic and apoptotic processes of myocardial cells, mesenchymal fibrotic and inflammatory reactions and of cytoskeleton and cellular matrix alterations making the myocardium more vulnerable [18] (Figure 5). Instead of decreasing cardiac work and oxygen request through the reduction of the afterload, the same compensatory hypertrophy of myocardial fibres induced by biomechanical stress seems to further damage cardiac performance. In remodelling myocardial muscle growth stimuli also activate biochemical signals that promote myocardiocytes apoptosis leading to pathologic hypertrophy with a negative effect on myocardial activity. This event is different from what normally happens in physiological hypertrophy of subjects doing physical activity, in whom biochemical signals that stimulate the myocardiocytes hypertrophy are associated with signals that promote their surviving [19] (Figure 6). An important aspect on myocardial remodelling is, above all, the modification of heart genetic expression that is characterized by the reactivation of fetal genetic program under the stimulation of some of the biochemical mediators released by myocardial fibres (adrenergic amines, endothelin, growth factors etc). Two events characterize these genetic modifications: a) re-expression of genes that were particularly active in fetal heart, such as the gene of the beta-myosin (molecule with low ATP-ase activity), some proto-oncogenes that induce apoptosis, such as C-Jun and C-Fos genes and the genes encoding for the α_1 _subunit of Na-K ATP-ase that can cause contractile dysfunction and instability of the membrane potential; b) suppression of genes that are active in adult heart, such as those regulating the sarcotubular ATP-ase, β_1_- adrenergic receptors and the lipid beta oxidation, with negative consequences on cardiac diastolic function and on the energetic metabolism. All these harmful events, connected to the cardiac remodelling, cause precarious heart function, and thus explain the fatal progressive evolution of chronic heart failure associated to AIC [20].

**Figure 4:**
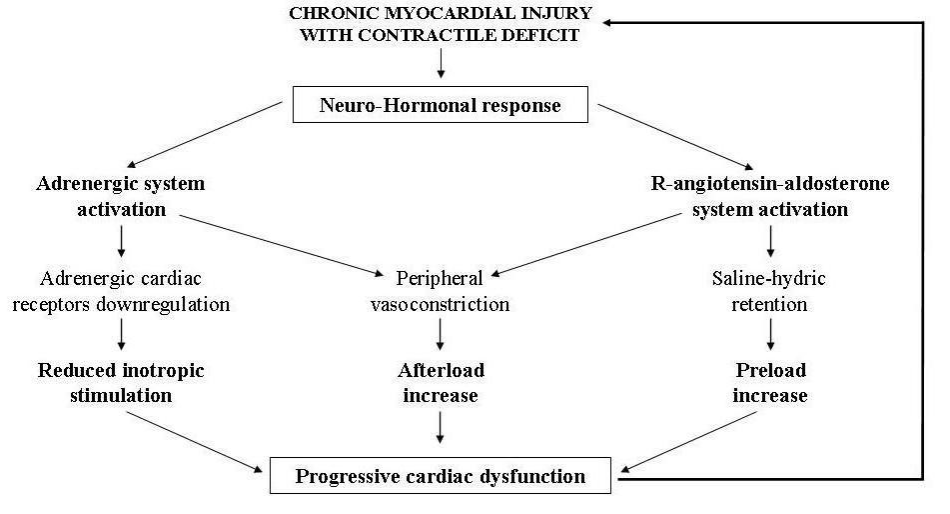
**Factors determining progression of cardiac insufficiency in anthracycline cardiomyopathy**.

**Figure 5:**
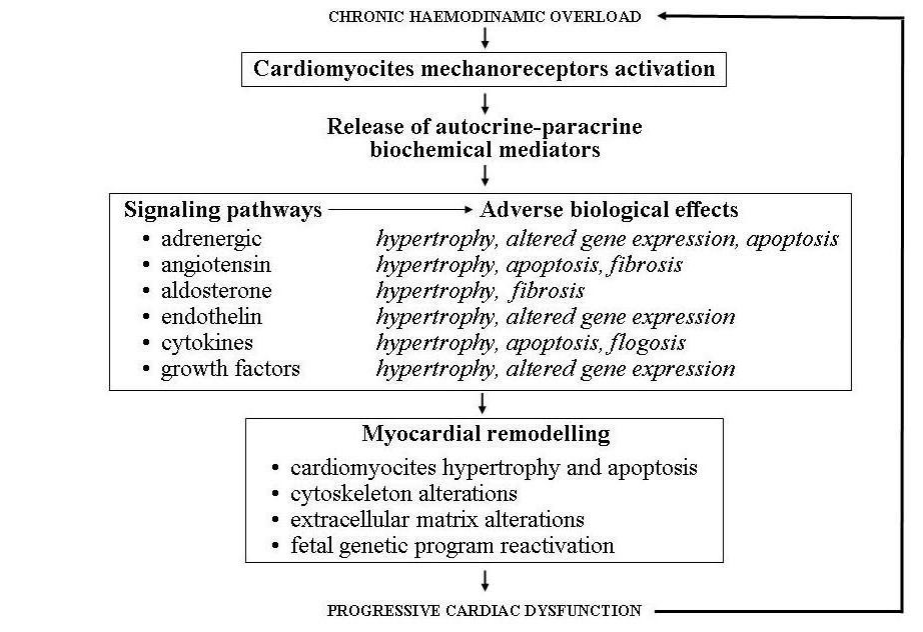
**Effects of intramyocardial neuro-hormonal response to biomechanic stress**.

**Figure 6:**
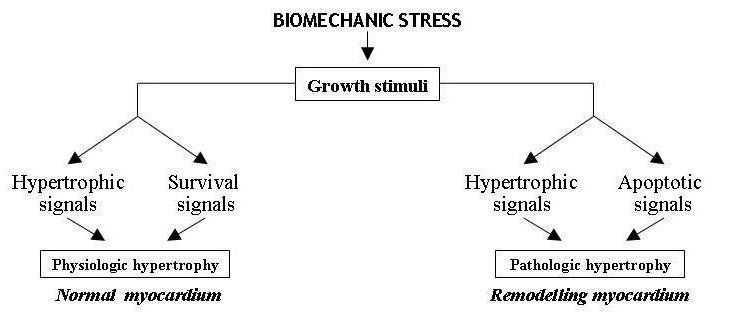
**Different to growth stimuli in myocardial remodelling**.

Prevention is particularly important in children, who, thanks to modern treatments, can survive leukemia and other tumoral diseases for several decades. The various approaches proposed are not always completely efficacious and include: cumulative dose under 450 mg/m^2^bs, use of anthracycline analogouses (epirubicin, idarubicin, mitoxantrone), alternative methods of administration (continuous slow infusion instead of rapid bolus, or liposome encapsulated anthracyclines) and, above all, use of antioxidants [3,6]. Although classic molecules such as tocopherol, ascorbic acid and acetylcysteine have displayed encouraging results against acute anthracycline toxicity, they have not demonstrated clear clinical benefits in chronic cardiomyopathty [6]. More recent studies reported that probucol, a lipid-lowering drug, that also exerts an antioxidant effect and promotes the activities of endogenous antioxidants, was effective in preventing anthracycline cardiomyopathty and heart failure in animal experiments, but further clinical trials are required [6]. To date the most promising agent is dexrarozane, an iron-chelator capable of preventing the formation of extremely reactive hydroxyl radicals catalyzed by the anthracycline-iron complex [21]. Clinical trials conducted in children have demonstrated that this drug has an effective cardioprotective action and reduces the cardiac side-effects of anthracyclines for up to 5 years after chemotherapy [22]. Longer follow-up are required to determine the long-term cardioprotective effects of dexrazozane.

The effectiveness of conventional therapy of chronic heart failure traditionally based on the use of digitalis, vasodilators, diuretics and beta-blockers is debated. Even if these drugs can transitorily improve the hemodynamic status of subjects with AIC, they are not able to prevent cardiac insufficiency progressing toward more severe forms requiring cardiac transplantation [23].

However, recent researches seem to offer new perspectives for pathogenetic treatment of AIC aimed at blocking the molecular mechanisms responsible for apoptotic, inflammatory and fibrotic phenomena connected to neuro-hormonal response causing heart remodelling, this being the key pathogenetic factor involved in the progression of chronic heart failure, regardless of its ethiology. Various treatments include the use of direct antagonists of angiotensin (losartan) and endothelin (bosentan), and of natriuretic peptides, physiologic antagonists of renine-angiotensine-aldosterone system. But the most promising seem to be based on the use of anticytokinic substances (monoclonal antibodies, soluble receptors) in particular those targeting tumour necrosis factor (TNF), or on the use of other substances that stimulate cardiomyocytes survival, such as growth factors (GH, IGF-1) and cardiotrophin, or those avoiding their apoptosis, such as caspases and metalloproteinases inhibitors [24-27]. These studies are still fragmentary, and the sometimes conflicting results need to be confirmed by larger clinical trials. Regarding AIC in particular, recent attention has been focused on some substances, such as Kinin B1 receptors (KB1R) antagonists and erythropoietin (Epo), that are able to module the function of AKT system which potentiates the biochemical signals connected to the survival of cardiomyiocytes at subcellular level and inhibits the mechanisms that stimulate apoptosis. Experimental studies in animals have shown that anthracyclines are capable of inducing the over expression of KB1R in cardiomyocytes, and this overexpression inhibiting the AKT pathway determines the appearance of apoptotic and inflammatory phenomena in the cardiac tissues. These negative effects in mice could be prevented by deleting the KB1R gene or by stimulating kinin B2 receptors that have a protective effect on cardiac muscle [28] (Figure 7). Regarding Epo, it has been shown that this molecule can directly stimulate the AKT biochemical system inside myocardial cells, hence promote the release of antiapoptotic, antioxidant and anti-inflammatory factors [29] (Figure 8). The results of these researches suggest that pharmacological antagonists of KB1R and Epo might be beneficial in AIC. Nevertheless, these potential therapeutic strategies have to be proven in further studies and has to be evaluated whether pharmacological KB1R antagonists and Epo can prevent the development of AIC or might even be curative when administered after the onset of the disease.

**Figure 7:**
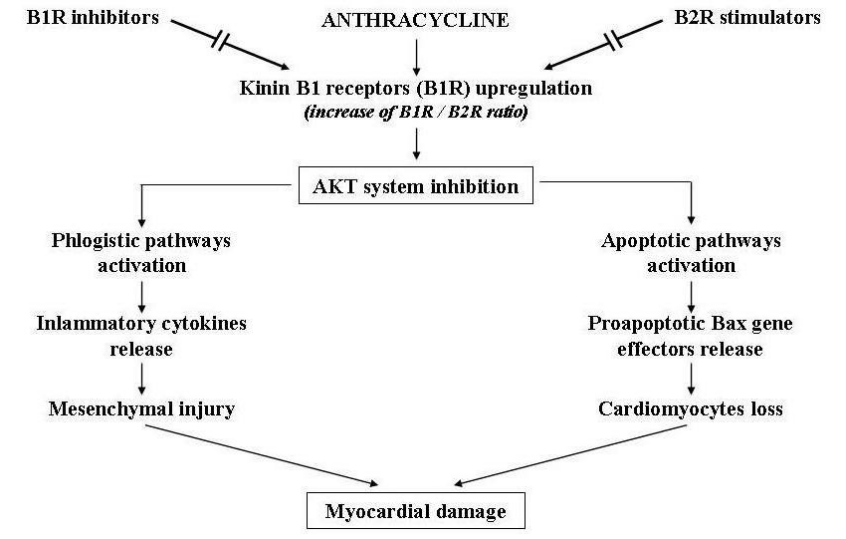
**Potential therapeutic use of B1 and B2 kinin receptors (B1R, B2R) modulators to prevent anthracycline-induced myocardial damage**.

**Figure 8:**
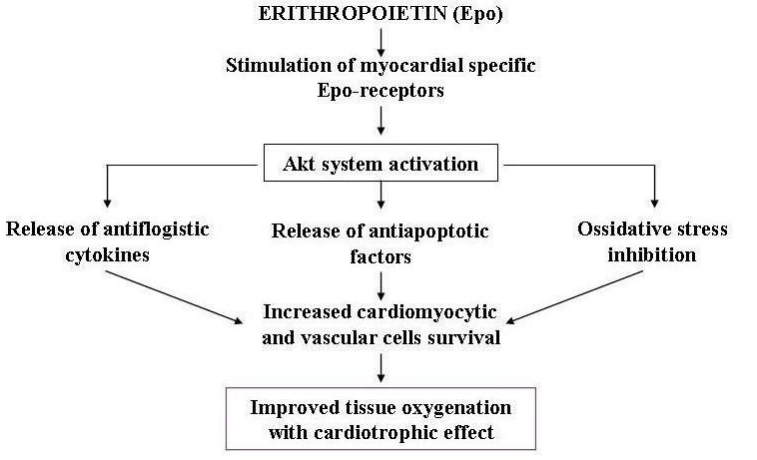
**Possible mechanism by which erythropoietin is cardioprotective**.

On the basis of these considerations, it is likely that in the near future the better knowledge of the subtle biochemical mechanisms regulating the function and survival of cardiac cells and the emerging perspectives of a "molecular ventricular assistance" connected to the developing gene therapy of chronic heart failure may allow a more rational preventive and therapeutic approach to cardiac insufficiency associated to dilated cardiomyopathies and therefore revolutionize also the prognosis of AIC [30,31].

## Competing interests

The author declares that they have no competing interests.
